# Dissociative symptoms are associated with reduced neuropsychological performance in patients with recurrent depression and a history of trauma exposure

**DOI:** 10.3402/ejpt.v7.29061

**Published:** 2016-02-25

**Authors:** Melissa Parlar, Paul A. Frewen, Carolina Oremus, Ruth A. Lanius, Margaret C. McKinnon

**Affiliations:** 1 Department of Psychiatry and Behavioral Neurosciences, McMaster University, Hamilton, Canada; 2 Mood Disorders Research Unit, St. Joseph's Healthcare, Hamilton, Canada; 3 Department of Psychiatry, University of Western Ontario, London, Canada; 4 Homewood Research Institute, Guelph, Canada

**Keywords:** cognition, depression, dissociation, transdiagnostic, traumatic stress

## Abstract

**Background:**

Although preliminary work suggests that dissociative symptoms may impact neuropsychological performance in trauma-exposed populations, the relation between dissociation and cognitive performance has not been explored in patients with depression.

**Objective:**

The present study examined dissociative symptoms in relation to neuropsychological performance in participants with a primary diagnosis of recurrent major depressive disorder (MDD) and a history of trauma exposure.

**Method:**

Twenty-three participants with MDD and 20 healthy controls who did not differ in age, sex, education, or IQ were assessed. In addition to a standardized neuropsychological battery assessing frontotemporally mediated cognitive processes, participants completed clinical measures assessing dissociative symptoms, illness severity, and past history of trauma exposure.

**Results:**

Among participants with MDD, greater severity of derealization was associated with reduced performance on measures of delayed visuospatial recall and recognition on a task of verbal memory recognition. In addition, more severe depersonalization was associated with slower processing speed and a response style lending itself toward better performance in a less active environment.

**Conclusions:**

These findings point toward dissociative symptoms as a transdiagnostic factor associated with neuropsychological dysfunction in patients with depression and a history of trauma. Limitations and recommendations for future research are discussed.

Cognitive dysfunction is widely reported among patients with major depressive disorder (MDD) (McDermott & Ebmeier, 2009; Rock, Roiser, Riedel, & Blackwell, 2014), contributing to impairments in multiple psychosocial domains (particularly employment) (McIntyre et al., 2013) and to disruptions in instrumental activities of daily living (Kiosses & Alexopoulos, 2005; McCall & Dunn, 2003). Critically, many persons with MDD have a comorbid history of trauma exposure, where in a sample of 2,000 participants with anxiety and/or depressive disorders, 91.2% reported experiencing a potentially traumatic or bothersome life event (Spinhoven, Penninx, van Hemert, de Rooij, & Elzinga, 2014). Trauma exposure and MDD have been linked to disruptions in a similar range of frontotemporally mediated cognitive functions, including recollective memory (Talarowska et al., 2010; Yehuda, Golier, Halligan, & Harvey, 2004), working memory (Gałecki et al., 2013; Vasterling et al., 2002), processing speed (Cohen et al., 2013; McDermott & Ebmeier, 2009), and cognitive flexibility (Polak, Witteveen, Reitsma, & Olff, 2012; Snyder, 2012).

It is unclear, however, whether symptoms common to trauma exposure and to depression contribute to this shared pattern of cognitive dysfunction. For example, dissociative symptoms are present in MDD and in trauma-related disorders (e.g., posttraumatic stress disorder [PTSD]) with some studies revealing a link between dissociation and cognitive functioning (De Bellis, Woolley, & Hooper, 2013; Roca, Hart, Kimbrell, & Freeman, 2006). Dissociation is described as “disruption of and/or discontinuity in the normal subjective integration of one or more aspects of psychological functioning, including—but not limited to—memory, identity, consciousness” (Spiegel et al., 2013). Etiologically, dissociation is thought to stem primarily from exposure to traumatic events or disruptions in attachment, serving to overmodulate affect and protect the individual from experiencing overwhelming distress during and/or after a traumatic event (Dalenberg et al., 2012; Lanius et al., 2010). Dissociative symptoms have been identified in persons with MDD, particularly among those with a history of trauma exposure. For example, in one study, compared to healthy controls, persons with MDD reported significantly higher levels of dissociative experiences (Molina-Serrano, Linotte, Amat, Souery, & Barreto, 2008). In the same study, those with a comorbid history of childhood trauma also tended to report higher levels of dissociative symptoms than those without a childhood trauma history (these results did not reach statistical significance). Increased HPA-axis reactivity and psychosocial stressors have also been related to dissociative symptoms in depressed persons (Bob, Fedor-Freybergh, Jasova, Bizik, et al., 2008), where dissociation (specifically dissociative disengagement) is thought to act as a defense mechanism related to a passive coping style (Bob, Fedor-Freybergh, Jasova, Susta, et al., 2008). Dissociation may be particularly related to more severe forms of depression, as depressed persons who also experience depersonalization tend to report more severe depressive symptoms (Žikić, Ćirić, & Mitković, 2009).

As noted, persons with depression as well as persons with PTSD show impaired performance on measures of frontotemporally mediated cognitive function, including executive functioning, verbal recollective memory (Johnsen & Asbjørnsen, 2009; Yehuda et al., 2004), attention (Vasterling, Brailey, Constans, & Sutker, 1998), and processing speed (Cohen et al., 2013). Here, recent meta-analyses further revealed a small-to-medium effect size on measures of executive functioning in PTSD (Polak et al., 2012) and small-to-large effect sizes on these measures in MDD (Snyder, 2012). Importantly, the presence of executive dysfunction has been found to impact negatively on both pharmacological (Dunkin et al., 2000) and non-pharmacological treatments (Wild & Gur, 2008) for affective disorders, where the ability to engage in and successfully complete treatment relies heavily on such processes (Johnco, Wuthrich, & Rapee, 2013; Kalayam & Alexopoulos, 1999).

To our knowledge, no studies have directly examined the extent to which dissociation contributes to cognitive dysfunction in MDD. This association has, however, begun to be elucidated in trauma-related disorders, where the presence of cognitive dysfunction among patients with dissociative symptoms may contribute to poorer treatment outcomes (Mansfield et al., 2010; Price, Kearns, Houry, & Rothbaum, 2014; Spitzer, Barnow, Freyberger, & Grabe, 2007) (see also Hagenaars, van Minnen, & Hoogduin, 2010; Halvorsen, Stenmark, Neuner, & Nordahl, 2014; Jaycox, Foa, & Morral, 1998). In one study, veterans with a comorbid dissociative disorder performed worse on measures of verbal recollective memory, attention, and executive functioning than veterans without a comorbid dissociative disorder (Roca et al., 2006). Further, higher levels of non-pathological dissociation among healthy subjects were associated with increased interference on a Stroop task (DePrince, Weinzierl, & Combs, 2008) and inhibitory deficits (Cromer, Stevens, DePrince, & Pears, 2006); these findings have been replicated in dissociative identity disorder (Dorahy, Middleton, & Irwin, 2005). Similarly, individuals with borderline personality disorder (BPD) who reported high levels of trait dissociation performed worse than healthy controls on tests of attention, executive functioning, and long-term memory (Haaland & Landrø, 2009). Higher levels of dissociation in BPD were also associated with a dampened limbic activation during an affective processing task (Krause-Utz et al., 2012). Further, healthy controls demonstrated episodic impairment for events encoded during an experimentally induced dissociative state (Bergouignan, Nyberg, & Ehrsson, 2014).

Here, we first compare self-reported levels of dissociation between patients with recurrent MDD and a history of trauma as compared to controls. We then examine the role of dissociation in cognitive functioning among patients with MDD as a potential transdiagnostic factor that could contribute to neuropsychological dysfunction observed across MDD and trauma-related disorders. We predicted that higher levels of depersonalization and derealization symptoms among persons with MDD and comorbid trauma exposure would be associated with lower scores on objective measures of frontotemporally mediated neurocognitive functions previously shown to be impacted in MDD.

## Method

### Participants

Twenty-three right-handed patients (mean age: 40.4 (14.2); 11 males, 12 females) who met DSM-IV diagnostic criteria for a primary diagnosis of recurrent (i.e., ≥3 episodes) MDD on the Structured Clinical Interview for DSM-IV-TR Axis I Disorders (SCID-I/P; First, Spitzer, Gibbon, & Williams, 2001) and who had a history of trauma exposure, according to responses on the Clinician-Administered PTSD Scale (CAPS; Blake et al., 1995) and/or Childhood Trauma Questionnaire (CTQ; Bernstein et al., 2003), were recruited. A control group consisted of 20 right-handed participants with no history of psychiatric illness or trauma exposure (mean age: 35.3 (14.1); 10 males, 10 females) who did not differ from the patient group in terms of sex distribution, age, education, or IQ. Among patients with MDD, five met criteria for moderate-to-severe childhood abuse on the CTQ only, nine met criteria for lifetime PTSD or trauma exposure on the CAPS only, and nine met criteria for childhood trauma exposure on the CTQ and a diagnosis of PTSD or trauma exposure on the CAPS. Among those participants who met criteria for PTSD or trauma exposure on the CAPS, 12 experienced interpersonal trauma (e.g., abuse by caregiver) and six experienced single-blow, accidental trauma (e.g., car accident).

Participants were recruited from St. Joseph's Healthcare Hamilton. Those with a past or current diagnosis of bipolar disorder, a psychotic disorder, neurological disease, traumatic brain injury and/or head injury with loss of consciousness (lasting more than 60 s), substance abuse in the past 6 months, current or lifetime history of substance dependence, and/or current or prior history of untreated significant medical illness were excluded. Participants were instructed not to use benzodiazepines within 12 hours prior to testing. The study was approved by the local Research Ethics Board and all participants provided written informed consent.

## Measures

### Clinical assessments

The Hamilton Rating Scale for Depression (HAM-D; Hamilton, 1960) was administered to assess the severity of depressive symptoms over the past week. We administered the CAPS to assess for current (i.e., past month) and past PTSD diagnostic status and symptom severity to confirm history of trauma exposure. Participants completed the CTQ to confirm a history of moderate-to-severe childhood trauma. The CTQ is a 28-item self-report questionnaire that measures (1) emotional abuse, (2) physical abuse, (3) sexual abuse, (4) emotional neglect, and (5) physical neglect. The CTQ has good internal consistency, and convergent reliability with therapist assessments of history of abuse is high (Bernstein et al., 2003). Finally, participants completed the Multiscale Dissociation Inventory (MDI; Briere, 2002). The MDI is a 30-item self-report questionnaire that assesses dissociative responses. The current study focuses on MDI depersonalization and derealization subscales, as these symptoms have been associated with increased depressive symptomatology (Molina-Seranno et al., 2008; Žikić et al., 2009) and form the basis of the recently described dissociative subtype of PTSD (Spiegel et al., 2013).

### Neuropsychological test battery

We administered an extensive battery of standardized neuropsychological measures aimed at measuring frontotemporally mediated cognitive functioning. *Current intellectual functioning*: (1) Wechsler Abbreviated Scale of Intelligence (WASI; Wechsler, 1999): one subtest of the performance index (i.e., matrix reasoning) and one subtest of the verbal index (i.e., vocabulary) were administered to calculate two-subtest full-scale IQ. *Declarative memory*: (1) California Verbal Learning Test-II (standard form) (CVLT-II; Delis, Kramer, Kaplan, & Ober, 2000): word learning task that provides indices of immediate and delayed memory performance, and recognition; (2) the Brief Visuospatial Memory Test-Revised (BVMT-R; Benedict, 1997) (Form 1): a nonverbal test of visuospatial memory under explicit encoding conditions. *Executive functioning*: (1) Color Trails Test (Parts 1 and 2) (D'Elia, Satz, Uchiyama, & White, 1996): measures attention, speed, and mental flexibility, including the ability to sequence two stimulus sets while alternating between them; (2) Wisconsin Card Sorting Test (128-item version) (WCST; Heaton, 2003): assesses the ability to form and shift concepts based on feedback. *Attention*: (1) Conners’ Continuous Performance Test—Second Edition (CPT-II; Conners, 2000): a computerized task measuring sustained attention and response inhibition.

### Statistical analysis

To examine group differences on demographic, clinical, and neuropsychological scores, two-tailed independent samples *t*-tests or Mann–Whitney U tests were performed, depending on whether data were normally distributed (as assessed using the Shapiro–Wilk test of normality).

MDI subscales were not normally distributed, and remained non-normal following log transformation. Associations between MDI subscale scores (depersonalization, derealization) and neuropsychological performance among the group with MDD and comorbid trauma exposure were therefore calculated using Spearman's rho (*r*_*s*_, two-tailed). The relation between normally distributed depressive symptoms (HAM-D scores) and neuropsychological performance was examined using Pearson correlation analyses. Significance was set at α=0.05 for all analyses. Analyses were conducted with SPSS 21 (IBM, Armonk, NY, USA).

## Results

### Demographic and clinical characteristics

Demographic and clinical data are displayed in Table 1. Age (*t*(41)=−1.18, *p*=0.24), education (*t*(41)=1.71, *p*=0.08), IQ (*t*(38)=1.49, *p*=0.15), and sex distribution (χ(1)= 0.03, *p=*0.85) did not differ significantly between groups. As expected, the MDD group had significantly higher scores on all clinical variables, including dissociative symptoms (e.g., HAM-D, CAPS, CTQ, and all MDI subscales).

**Table 1:** Demographic and clinical characteristics of the study sample

Characteristics	MDD with trauma (*n*=23)	Healthy controls (*n*=20)
	Mean (SD)	Mean (SD)
*Demographic characteristics*		
Age	40.4 (14.2)	35.3 (14.1)
Sex (female: male)	12:11	10:10
IQ	114.0 (13.7)	120.2 (12.5)
Years of education	15.2 (3.7)	16.9 (2.4)
*Clinical characteristics*		
HAM-D	**11.7 (5.9)	0.5 (0.8)
CAPS (month)	**32.2 (33.2)	0.0 (0.0)
Childhood Trauma Questionnaire		
Emotional abuse	**13.3 (6.4)	6.5 (1.3)
Physical abuse	**7.5 (2.8)	5.5 (0.8)
Sexual abuse	**7.5 (5.4)	5.0 (0.0)
Emotional neglect	**13.7 (5.4)	8.1 (3.0)
Physical neglect	**9.1 (5.1)	5.6 (0.8)
MDI (total)	**51.1 (15.6)	33.8 (2.9)
Disengagement	**12.7 (3.9)	8.1 (2.1)
Depersonalization	**7.5 (2.6)	5.0 (0.3)
Derealization	**7.7 (3.6)	5.3 (0.6)
Emotional constriction	**9.4 (5.1)	5.2 (0.8)
Memory disturbance	**8.3 (4.1)	5.4 (0.7)
Identity dissociation	*5.6 (1.7)	5.0 (0.0)
Number of depressive episodes	13.2 (14.9)	NA
Age of MDD onset	20.6 (10.1)	NA

Abbreviations: CAPS, Clinician-Administered PTSD Scale; HAM-D, Hamilton Depression Rating Scale; MDD, major depressive disorder; MDI, Multiscale Dissociation Inventory.

* *p*<0.05,

** *p*<0.01

### Neuropsychological performance

Group comparisons of performance on neuropsychological tests are displayed in Table 2. Participants with MDD and trauma exposure performed significantly worse than controls on several neuropsychological measures. Those with MDD recalled significantly fewer words on the CVLT-II Long-Delay Free Recall condition, based on both raw and *z*-scores (*U*=141, *z*=−2.19, *p*=0.03, *r*=−0.33; *U*=145, *z*=−2.11, *p*=0.035, *r*=−0.32, respectively). On the same measure, MDD participants made fewer hits on the Recognition trial compared to controls, based on both raw and *z*-scores (*U*=140, *z*=−2.31, *p=*0.021, *r=*−0.35; *U*=136, *z*=−2.37, *p*=0.018, *r*=−0.36, respectively). Participants with depression also performed worse than controls on Color Trails Test Part 2 completion time (raw scores), a measure of mental flexibility and processing speed (*U*=100, *z*=−2.81, *p*=0.005, *r*=−0.44).

**Table 2:** Raw and standardized scores on measures of neuropsychological performance

Cognitive variable	MDD with trauma (*n*=23)		Healthy controls (*n*=20)	
	
	Raw score, mean (SD)	*t-*score, mean (SD)	Raw score, mean (SD)	*t*-score, mean (SD)
**CVLT-II**				
Trials 1–5 total	52.1 (9.0)	52.0 (9.9)	56.0 (9.8)	55.7 (10.3)
Short-delay free recall (z)	11.4 (2.5)	0.17 (0.5)	12.5 (3.3)	0.50 (1.1)
Long-delay free recall (z)	*11.4 (2.5)	*−0.08 (0.5)	*13.2 (2.8)	*0.50 (1.1)
Recognition hits (z)	*14.6 (1.4)	*−0.48 (0.8)	*15.4 (0.9)	*0.05 (0.6)
False positives (z)	0.9 (1.1)	0.15 (0.7)	0.7 (1.5)	0.18 (0.6)
**BVMT-R**				
Trials 1–3 total	25.9 (5.8)	50.5 (10.3)	27.0 (6.4)	51.1 (11.8)
Delayed recall	10.7 (1.6)	55.8 (8.3)	10.4 (1.5)	52.1 (9.4)
**Color Trails Test**				
Part 1	31.8 (12.9)	54.6 (9.0)	26.8 (7.8)	54.8 (5.8)
Part 2	**66.5 (20.2)	56.4 (7.5)	**51.0 (13.9)	59.2 (5.2)
**WCST**				
Total errors	22.9 (18.0)	48.7 (8.8)	19.9 (20.3)	49.5 (9.7)
Perseverative errors	11.7 (11.4)	50.2 (9.8)	11.1 (11.1)	48.5 (10.1)
**CPT-II**				
Omissions	3.4 (12.1)	54.8 (40.5)	1.6 (3.0)	47.3 (8.9)
Commissions	14.1 (7.9)	52.5 (11.8)	11.7 (5.4)	50.2 (9.0)
Hit Reaction Time Block Change	−0.001 (0.02)	48.3 (7.8)	−0.006 (0.01)	47.1 (5.4)
Hit Reaction Time ISI	0.03 (0.03)	46.5 (12.1)	0.04 (0.02)	46.1 (6.9)

Abbreviations: BVMT-R, Brief Visuospatial Memory Test-Revised; CVLT-II, California Verbal Learning Test-II; CPT-II, Conners’ Continuous Performance Test-II; WCST, Wisconsin Card Sorting Test. *Note*: T-score conversions used for neuropsychological test scores unless otherwise stated (e.g. “(z)” denotes *z*-scores).

* *p*<0.05,

** *p*<0.01

### Correlations between neuropsychological performance and dissociative and depressive symptoms

Results of correlation analyses conducted among the group with MDD and trauma exposure using standardized scores on the neuropsychological measures (e.g., *t*- and *z*-scores) are reported in Table 3. Significant correlations emerged between MDI derealization symptoms and measures of verbal and visuospatial memory. Specifically, derealization symptoms were positively correlated with CVLT-II False Positive *z*-scores (*r*_*s*_=0.43, *p*=0.04), where higher *z*-scores reflect a higher number of false positives. Derealization symptoms were also negatively associated with BVMT-R Delayed Recall *t*-scores (*r*_*s*_=−0.57, *p*=0.004), where lower *t*-scores reflect worse performance on delayed recall. In addition, MDI depersonalization scores were correlated with measures of processing speed (and mental flexibility) and sustained attention. Specifically, Color Trails Test Part 2 *t-*scores were negatively correlated with depersonalization symptoms (*r*_*s*_=−0.42, *p*=0.047), where lower t-scores indicate slower completion time. MDI depersonalization was also associated with CPT-II Hit Reaction Time (RT) Interstimulus Interval (ISI) Change *t*-scores (vigilance measure) (*r*_*s*_=−0.42, *p*=0.05) (lower *t*-scores indicate sustained or increased response speed when stimuli are presented more slowly). In striking contrast, not a single significant correlation emerged between depressive symptom severity (HAM-D scores) and variability in neuropsychological performance.

**Table 3:** Correlations between dissociative symptoms, depressive symptoms, and standardized scores on neuropsychological measures

Cognitive variable	*ρ* MDI Depers.	*ρ* MDI Dereal.	*r* HAM-D
**CVLT-II**			
Trials 1–5 total	+0.11	−0.27	−0.23
Short-delay free recall (z)	+0.31	−0.01	−0.02
Long-delay free recall (z)	+0.31	+0.23	+0.05
Recognition hits (z)	+0.14	−0.01	+0.08
False positives (z)	−0.22	*+0.43	−0.11
**BVMT-R**			
Trials 1–3 total	−0.05	−0.40	−0.19
Delayed Recall	−0.22	**−0.57	−0.17
**Color Trails Test**			
Part 1	−0.22	−0.05	+0.07
Part 2	*−0.42	−0.21	−0.31
**WCST**			
Total errors	0.01	−0.11	−0.18
Perseverative errors	0.07	−0.05	+0.08
**CPT-II**			
Omissions	−0.04	+0.28	+0.39
Commissions	−0.12	+0.06	−0.38
Hit Reaction Time Block Change	+0.28	+0.22	+0.07
Hit Reaction Time ISI	*−0.42	−0.36	+0.26

Abbreviations: BVMT-R, Brief Visuospatial Memory Test-Revised; CVLT-II, California Verbal Learning Test-II; CPT-II, Conners’ Continuous Performance Test-II; HAM-D, Hamilton Rating Scale for Depression; MDI; Multiscale Dissociation Inventory; RT, reaction time; SE, standard error; WCST, Wisconsin Card Sorting Test. Values denoted by *ρ* indicate Spearman's correlation coefficient. Values denoted by *r* indicate Pearson's correlation coefficient. *Note*: T-score conversions used for neuropsychological test scores unless otherwise stated (e.g., “z”). Higher *z*-scores on CVLT-II False Positive scale indicate worse performance.

* *p*<0.05,

** *p*<0.01

## Discussion

To the best of our knowledge, this is the first study to examine the relation between dissociative symptoms and neuropsychological performance in participants with a primary diagnosis of recurrent MDD. Despite a relatively small sample size and heterogeneity across persons with respect to history of trauma exposure, our findings indicate that persons with recurrent MDD and a history of trauma report significantly higher levels of dissociation compared with controls, an effect that was seen across each of the subscales of the MDI. Dissociative phenomena, therefore, should not be overlooked in depressed persons. Moreover, the main findings also point toward a detrimental association between dissociative symptoms and neuropsychological performance in depressed persons, particularly in frontotemporally mediated domains of cognition (i.e., memory and attention). The findings of the current study suggest that higher levels of dissociative symptoms (specifically derealization), rather than depression symptom severity, are related to worse performance on measures of verbal memory and visuospatial memory. Moreover, higher levels of depersonalization, but not depressive symptoms, were related to poorer processing speed, as well as a response style on a task of sustained attention indicative of faster reaction time when stimuli are presented more slowly. These findings suggest that greater dissociation is associated with higher vigilance in less active environments.

The link between dissociative symptoms and poor verbal declarative memory has been documented in healthy participants who were classified as high versus low dissociators (Amrhein, Hengmith, Maragkos, & Hennig-Fast, 2008), veterans with PTSD and dissociative disorder (Roca et al., 2006), psychiatric inpatients (Prohl, Resch, Parzer, & Brunner, 2001), and patients with depersonalization disorder (Guralnik, Schmeidler, & Simeon, 2000). Although there were no associations between dissociative symptoms and verbal learning or delayed recall scores on the CVLT-II in our MDD group, there was a signification correlation with scores on the recognition trial of this task. Specifically, higher levels of derealization were related to more false-positive recognition errors. These results could be reflective of a negative impact of derealization on verbal learning and memory, but may also be related to increased suggestibility. Indeed, high levels of dissociation have been shown to predict a greater tendency to incorporate misleading information into memory (Eisen & Carlson, 1998). In our sample, it appears that being more prone to derealization is associated with an increased likelihood to think that a stimulus being presented for the first time has in fact already been presented.

Our finding that higher levels of derealization are associated with worse delayed visuospatial recall on the BVMT-R is in line with findings by Morgan, Doran, Steffianm, Hazlett, and Southwick (2006) showing that in a group of special operations soldiers, higher levels of dissociation were associated with worse recall on the Rey–Osterrieth Complex Figure (ROCF). A similar relation has been demonstrated in healthy participants following experimentally inducted dissociation, who exhibited worse ROCF recall as compared to a control group (Brewin & Mersaditabari, 2013). Persons with depersonalization disorder also showed poorer performance on visuospatial tasks of the Wechsler Memory Scale (Guralnik et al., 2000). However, Haaland et al. (2009) failed to find an effect for dissociation in persons with BPD on a task of nonverbal memory.

Among the MDD group, there were also associations between dissociation and attention. Higher levels of depersonalization were related to slower completion time on the attention and processing speed task, Color Trails Test, Part 2. In comparison, no correlations emerged between Color Trails Test, Part 1 and dissociative symptoms. These results are consistent with findings by Roca et al. (2006) among a sample of veterans with PTSD and a dissociative disorder. In addition to measuring attention, Part 2 of the Color Trails Test also assesses mental flexibility, a domain of executive functioning. Other studies have examined the link between executive function and dissociation and have found that impairments in executive functioning, as measured by a random number generation task and by the Behavioural Assessment of Dysexecutive Syndrome, were related to dissociation (Horne, Evans, & Orne, 1982; Wilson, Alderman, Burgess, Emslie, & Evans, 1996) (see also Bruce, Ray, Bruce, Arnett, & Carlson, 2007 for conflicting results).

Depersonalization symptoms on the MDI were also related to the CPT-II RT ISI Change *t*-score variable (a measure of vigilance), such that higher levels of depersonalization were related to better performance in a less active environment (where stimuli were presented less frequently). These results are in line with other studies that suggest that, depending on the attentional condition, dissociation may actually result in improved performance. For example, higher levels of dissociation are related to better interference control under divided attention conditions (e.g., on the Stroop task) and worse interference control under selective attention conditions (DePrince, Weinzierl, & Combs, 2008). These results may be explained by the cognitive environments conceptualization of dissociation (DePrince & Freyd, 1999), which suggests that dissociation may coincide with chronically fragmented attention, resulting in the ability to process multiple information streams simultaneously. Other studies, however, point toward attentional deficits caused by dissociation. Specifically, in children with a history of intrafamilial sexual abuse, dissociation mediates the relation between PTSD symptoms and attentional problems (Kaplow, Hall, Koenen, Dodge, & Amaya-Jackson, 2008). Similar evidence suggests that higher levels of dissociation among maltreated children are related to poorer performance on measures of attention (De Bellis et al., 2013).

The current findings point toward a number of important avenues for further investigation. For example, future studies may examine the role of dissociation in cognitive performance as a function of trauma exposure subtypes and/or disrupted attachment patterns and should compare neuropsychological performance in persons with MDD with and without a history of trauma exposure. Dissociative symptoms may be more severe in those with a history of sexual abuse, compared to those with a history physical abuse or combined physical and sexual abuse (Boysan, Goldsmith, Cavuş, Kayri, & Keskin, 2009). The role of attachment patterns should be considered in future studies. In one longitudinal study, the quality of caregiving during childhood was the strongest predictor of dissociative symptoms in adulthood, whereas extent of abuse was not predictive (Dutra, Bureau, Holmes, Lyubchik, & Lyons-Ruth, 2009).

## Conclusions

The findings of this study could have implications for clinicians and researchers working with depressed persons, and particularly those with a history of trauma exposure. Assessment of dissociative symptoms is rarely undertaken in those with MDD.

Our results are in line with the previous findings, which suggest that dissociation is related to specific and subtle impairments in neurocognition (Giesbrecht, Lynn, Lilienfeld, & Merckelbach, 2008). Given these findings, dissociative symptoms should ideally be assessed pretreatment, as their effect on cognition may impact MDD treatment response. Given that a dissociative subtype of PTSD marked by increased depersonalization and derealization was included in the most recent version of the DSM-5 (APA, 2013), it may be reasonable to suspect that a dissociative subtype may be likewise present in a subset of persons with MDD. The findings of the present study point toward the urgent need to further investigate the impact of dissociation on neurocognition and other domains of functioning in participants with depression and a history of trauma exposure.
